# Evidence for the critical role of transmembrane helices 1 and 7 in substrate transport by human P-glycoprotein (ABCB1)

**DOI:** 10.1371/journal.pone.0204693

**Published:** 2018-09-28

**Authors:** Andaleeb Sajid, Sabrina Lusvarghi, Eduardo E. Chufan, Suresh V. Ambudkar

**Affiliations:** Laboratory of Cell Biology, Center for Cancer Research, National Cancer Institute, National Institutes of Health, Bethesda, Maryland, United States of America; University of Cambridge, UNITED KINGDOM

## Abstract

P-glycoprotein (P-gp) is an ABC transporter that exports many amphipathic or hydrophobic compounds, including chemically and functionally dissimilar anticancer drugs, from cells. To understand the role of transmembrane helices (TMH) 1 and 7 in drug-binding and transport, we selected six residues from both TMH1 (V53, I59, I60, L65, M68 and F72) and TMH7 (V713, I719, I720, Q725, F728 and F732); and substituted them with alanine by gene synthesis to generate a variant termed “TMH1,7 mutant P-gp”. The expression and function of TMH1,7 mutant P-gp with twelve mutations was characterized using the BacMam baculovirus-HeLa cell expression system. The expression and conformation of TMH1,7 mutant P-gp was not altered by the introduction of the twelve mutations, as confirmed by using the human P-gp-specific antibodies UIC2, MRK16 and 4E3. We tested 25 fluorescently-labeled substrates and found that only three substrates, NBD-cyclosporine A, Rhod-2-AM and X-Rhod-1-AM were transported by the TMH1,7 mutant. The basal ATPase activity of TMH1,7 mutant P-gp was lower (40–50%) compared to wild-type (WT) P-gp, despite similar level of expression. Although most of the substrates modulate ATPase activity of P-gp, the activity of TMH1,7 mutant transporter was not significantly modulated by any of the tested substrates. Docking of selected substrates in homology models showed comparable docking scores for the TMH1,7 mutant and WT P-gp, although the binding conformations were different. Both the ATPase assay and *in silico* docking analyses suggest that the interactions with residues in the drug-binding pocket are altered as a consequence of the mutations. We demonstrate that it is possible to generate a variant of P-gp with a loss of broad substrate specificity and propose that TMH1 and TMH7 play a critical role in the drug efflux function of this multidrug transporter.

## Introduction

The treatment of several cancer types is hindered by development of drug-resistant forms. In many cases, cancer cells develop drug resistance due to over-expression of P-glycoprotein (P-gp, ABCB1), which is an ATP-Binding Cassette (ABC) transporter involved in the efflux of drugs from cells, thereby reducing their intracellular concentrations [1–4]. The polyspecificity of P-gp enables it to export a wide range of chemically dissimilar compounds that are either amphipathic or hydrophobic [5–7]. P-gp is a highly conserved membrane protein present throughout eukaryotic species. In humans, it is expressed by epithelial cells of the intestine, kidney, liver, placenta, adrenal gland and by endothelial cells at blood-brain barrier. The major function of P-gp is exporting toxins and xenobiotics from cells, protecting them from the harmful effects of these compounds [5, 8–10]. Thus, P-gp plays a role in the availability and pharmacokinetics of several drugs.

Human P-gp is comprised of twelve transmembrane helices (TMHs) divided into two homologous halves. The N-terminal half is comprised of transmembrane domain 1 (TMD1) and nucleotide-binding domain 1 (NBD1). Similarly, the C-terminal half is comprised of TMD2 and NBD2. Each TMD contains six transmembrane helices (TMH) joined by extracellular loops (ECLs) and intracellular loops (ICLs). The NBDs carry out ATP binding and hydrolysis, which facilitates the transport of substrates [1, 11–14]. Hence, most substrates stimulate its ATPase activity [15–17]. During the transport cycle, P-gp alternates between inward-facing (inverted V-shape) and outward-facing conformations. The crystal structure of mouse P-gp in the inward-facing conformation has been reported in multiple studies that have revealed the location of TMHs, NBDs, ECLs and ICLs [18–21]. The mouse P-gp structures were used as a template for modeling studies of human P-gp. Recently, a high-resolution cryo-EM structure of human P-gp (ATP-bound E-Q mutant) was reported, which is the first study showing the outward-facing conformation [22], thus demonstrating that there are at least two major conformations of P-gp.

Despite numerous studies, the mechanisms of P-gp transport and conformational changes are not yet well characterized. To understand the transport mechanism and molecular basis of P-gp polyspecificity, several single, double or triple mutations of residues in the drug-binding pocket have been studied [17, 23–28]. Within its large drug-binding pocket, there are almost forty residues involved in binding and transport; therefore, P-gp generally does not lose the ability to transport substrates due to mutations in a few residues of the pocket. However, mutations in the NBDs do abrogate P-gp activity [22, 29]. A number of studies have shown the existence of overlapping binding sites for different substrates as well as multiple binding sites for a given substrate, indicating the involvement of multiple residues within the drug-binding pocket [17, 23, 30–33]. In a recent study, we generated a mutant of P-gp termed 15Y with fifteen conserved residues mutated to tyrosine to determine the extent of flexibility of the drug-binding pocket [16]. The 15Y mutant was stably expressed and was able to transport most of the small and medium-size substrates tested, although the substrates with large molecular weight (>1000 Da) were not transported, demonstrating the extent of flexibility of the drug-binding pocket of human P-gp.

In this study, we address the role of TMH1 and TMH7 in binding and transport of substrates. TMH1 and TMH7 are topologically identical helices in the two halves of P-gp and may together regulate substrate transport. We selected six conserved residues each from TMH1 and TMH7 and substituted them with alanine to generate TMH1,7 mutant P-gp. We tested the ability of TMH1,7 mutant P-gp to transport a variety of fluorescent substrates that are chemically and structurally distinct. We found that TMH1,7 mutant P-gp was expressed normally in HeLa cells but was only able to transport three of the 25 substrates tested. We also observed a partial loss of vanadate-sensitive basal ATPase activity and lack of stimulation or inhibition by the tested substrates. *In silico* docking of substrates in the homology models of the TMH1,7 mutant and WT showed that even though no significant difference was found for the docking scores, the specific interactions within the drug-binding pocket changed due to the mutations. Our findings strongly suggest a critical role for TMH1 and TMH7 in the transport function of human P-gp. In addition, we describe the generation of mutant P-gp with a very narrow substrate specificity.

## Materials and methods

### Chemicals and antibodies

The fluorescent compounds tetramethylrosamine chloride (TMR-Cl), BODIPY (BD)-verapamil, 3-propionyl ethylenediamine hydrochloride (BD-EDA), BD-prazosin, BD-vinblastine, tetramethylrhodamine ethyl ester perchlorate (TMRE), 3,3'-diethyloxacarbocyanine iodide (DiOC_2_), quinolinium 6-(dimethylamino)-2-[4-[4-(dimethylamino)phenyl]-1,3-butadienyl]-1-ethyl perchlorate (LDS-751), SYTO13, rhodamine 123, calcein-AM, dihydrorhodamine 123, Mito-tracker deep red FM, ER tracker red, Rhod-2-AM, X-Rhod-1-AM, tetramethylrhodamine methyl ester perchlorate (TMRM), rhodamine B hexyl ester were purchased from Invitrogen/ThermoFisher (Carlsbad, CA). Flutax-1 was purchased from Tocris biosciences (Minneapolis, MN). Cyclosporine A was purchased from the Alexis Corporation (Lausen, Switzerland). NBD-cyclosporine A was generously provided by Drs. Anika Hartz and Björn Bauer, University of Kentucky (Lexington, KY). All remaining chemicals were purchased from Sigma-Aldrich (St. Louis, MO).

The P-gp specific monoclonal antibody C219 was provided by Fujirebio Diagnostic Inc. (Malvern, PA), and the MRK16 antibody was purchased from Kyowa Medex Company (Tokyo, Japan). 4E3 antibody was purchased from Abcam (Cambridge, MA) and UIC2 was purchased from eBioscience (San Diego, CA) [17]. FITC-labeled anti-mouse secondary antibody IgG2aκ isotype was obtained from BD Biosciences (San Jose, CA).

### Cell lines and culture conditions

HeLa cells were purchased from American Type Culture Collection (ATCC, Manassas, VA, USA). Cells were maintained in Dulbecco’s modified Eagle’s Medium (DMEM, Difco) supplemented with 10% Fetal Bovine Serum, 5 mM L-glutamine, 100 units/mL penicillin and 100 μg/mL streptomycin at 37°C in 5% CO_2_.

### Generation of TMH1,7 mutant P-gp

The residues in TMH1 and TMH7 were selected as described in the results section. Selected residues (V53, I59, I60, L65, M68, F72 in TMH1 and V713, I719, I720, Q725, F728, F732 in TMH7) were substituted with alanine in human P-gp sequence using the GeneArt gene synthesis method (Life technologies, ThermoFisher).

### Recombinant BacMam and baculovirus generation

The Bac-to-Bac Baculovirus Expression System (Life Technologies, Carlsbad, CA) was used to generate recombinant baculovirus and BacMam baculovirus, as described previously [17, 34]. Briefly, WT and mutant *MDR*1 genes were cloned in pDonr-255 and used for Gateway cloning into pDest-625 (HeLa cell expression) and pDest-008 (insect cells expression). pDest clones were used to generate the Bacmids for generation of BacMam and baculovirus following the manufacturer’s protocol (Gibco, ThermoFisher).

### BacMam baculovirus transduction of HeLa cells and expression of wild-type and TMH1,7 mutant P-gp

HeLa cells were transduced with the WT or TMH1,7 mutant P-gp BacMam baculovirus at a titer of 30–100 virus particles per cell, as described previously [16, 17, 35]. Twenty-two hours post-transduction, the cells were trypsinized and harvested. HeLa cells (3x10^5^/tube) were analyzed for cell surface expression of WT and TMH1,7 mutant P-gp by incubation with the human P-gp specific monoclonal antibodies MRK16 (1 μg per 100,000 cells), 4E3 (0.5 μg per 100,000 cells), UIC2 (2 μg per 100,000 cells) and mouse IgG2aκ antibody control (1 μg per 100,000 cells) for 30 min in Iscove's Modified Dulbecco's Medium (IMDM, Difco) containing 5% FBS at 37°C. Cells were washed with cold IMDM and incubated with FITC-labeled anti-mouse secondary antibody IgG2aκ (0.25 μg per 100,000 cells) at 37°C. After incubation, cells were washed with cold IMDM and re-suspended in cold PBS containing 1% BSA. The stained cells were analyzed by flow cytometry using a FACS CANTO II instrument with BD FACSDiva software (BD biosciences), and the data were analyzed using FlowJo software (Tree Star, Inc. Ashland, OR).

### Transport of fluorescent substrates

For transport assays of WT and TMH1,7 mutant P-gp, 3x10^5^ HeLa cells in IMDM medium containing 5% FBS were incubated with fluorescent substrates at final concentrations as follows: calcein-AM, TMRM, TMRC, BD-verapamil, BD-prazosin, DiOC_2_, JC-1, SYTO13, LDS-751, azide-fluor 545, NBD-cyclosporine A, ER Tracker, Mito-tracker, rhodamine B hexyl ester, Rhod-2-AM, X-Rhod-1-AM and BD-vinblastine at 0.5 μM; daunorubicin at 4 μM; flutax-1 at 5 μM; rhodamine 123 and dihydrorhodamine 123 at 1.3 μM; BD-EDA, TMRE, rhodamine 6G and dihydrorhodamine 6G at 1 μM. Cells were incubated with all substrates for 45 min (except with calcein-AM for 10 min) at 37°C. After incubation, cells were washed with cold IMDM and re-suspended in cold PBS containing 1% BSA. The transport of substrates was measured by flow-cytometry using untransduced cells as a control. The mean fluorescence intensity of WT P-gp-expressing cells after subtraction from that of untransduced cells was taken as 100% efflux. Transport efficacy of TMH1,7 mutant P-gp was also calculated similarly and expressed as percent of WT. All substrates were tested three or more times and the results were analyzed as described in the previous section.

### Preparation of total membranes from High Five insect cells

High Five insect cells (Invitrogen, Carlsbad, CA) were infected with recombinant baculovirus carrying WT or TMH1,7 mutant P-gp with a 6X His-tag and TEV-cleavage site at the C-terminal end, as described previously [14, 34, 36]. Membrane vesicles were prepared by hypotonic lysis of P-gp-expressing High Five insect cells and differential centrifugation [37, 38].

### SDS-PAGE and Western blotting

HeLa cells (5X10^5^) expressing WT or TMH1,7 mutant P-gp were lysed by sonication and freeze-thaw cycles in a buffer containing 10 mM Tris-Cl pH 8.0, 0.1% Triton X-100, 10 mM MgSO_4_, 2 mM CaCl_2_, 1% aprotinin, 1 mM AEBSF, 2 mM DTT and 20 μg/mL nuclease. Samples (lysates of 60,000 cells) were resolved by 7% Tris-acetate SDS-PAGE gels and Western blotting was done using C219 (anti-P-gp, 1:2,000 dilution) and GAPDH-6C5 (1:30,000 dilution) monoclonal antibodies (primary) and HRP-conjugated mouse IgG secondary antibody (1:10,000 dilution). The blots were developed using an ECL western blotting detection kit (GE healthcare, Pittsburgh, PA). The quantitation of blots was done by FiJi-ImageJ. For P-gp expression in insect cells, membrane vesicles (10 μg total protein/lane) with the WT and TMH1,7 mutant P-gp were resolved by 7% Tris-acetate SDS-PAGE and stained with colloidal blue. Gels were scanned using Licor Odyssey CLx ImageStudio software. For Western blotting with the P-gp-specific C219 antibody, a similar procedure as above was followed using insect cell membranes (1 μg total protein/lane). For quantification, each SDS-PAGE and immunoblotting experiment was repeated at least three times and values represent mean ± SD.

### ATPase assay

ATP hydrolysis was measured in membrane vesicles of High Five insect cells expressing WT or TMH1,7 mutant P-gp. Membranes (10 μg protein per 100 μL reaction volume) were incubated in the presence or absence of 0.3 mM sodium orthovanadate in ATPase assay buffer (50 mM MES-Tris pH 6.8, 50 mM KCl, 10 mM MgCl_2_, 5 mM NaN_3_, 1 mM EGTA, 1 mM ouabain, and 2 mM DTT). Basal ATPase activity was measured in the presence of DMSO, while drug-modulated activity was measured in the presence of selected drugs (drug stocks prepared at 100X concentration in DMSO). The reaction was started at 37°C by addition of 5 mM ATP and stopped by addition of 2.5% SDS after 20 min incubation. The level of generated inorganic phosphate was quantified with a colorimetric method [37–39]. The vanadate-sensitive ATPase activities were analyzed and plotted with GraphPad Prism software (version 7).

### *In silico* analysis

A homology model for human P-gp was generated using the sequence of the human P-gp (accession # UniprotKB: P08183) or the TMH1,7 mutant and the mouse P-gp structure (PDB.5KPI, 87% protein sequence identity, [21]) as a template for the inward-facing conformation. For the outward-facing conformation, the structure of the ATP-bound E-Q mutant of human P-gp (PDB.6C0V) was used [22]. SWISS-MODEL (https://swissmodel.expasy.org/) [40] was used to generate models of both the WT and TMH1,7 mutant P-gp. Molecular models were analyzed using Pymol [41, 42].

The docking studies were performed as described previously [17]. Briefly, the MGL tools software package [43] was used to prepare the receptor and ligand structures. AutoDock Vina [44] was used for flexible receptor docking using the substrates given in S2 Table. The residues that were found to interact with ligands QZ59-RRR and QZ59-SSS in the mouse P-gp X-ray structure were selected to be used as flexible side-chains for docking [19]. These residues are L65, M68, M69, F72, Q195, F303, I306, Y307, F336, L339, I340, F343, Q347, N721, Q725, F728, F732, M949, Y953, F957, L975, F978, V982, M986, Q990, and S993. Additionally, V53, I59, I60, V713, V719 and V720 were included among the flexible side-chain residues, so all the mutated amino acids were flexible. The receptor grid was defined in the center at x = 20, y = 55 and z = 5. The inner box dimensions were 70x40x40 Å and the exhaustiveness level was set at 100.

## Results

### Rationale for the selection of residues in TMH1 and TMH7 for mutagenesis

**Fig 1:**
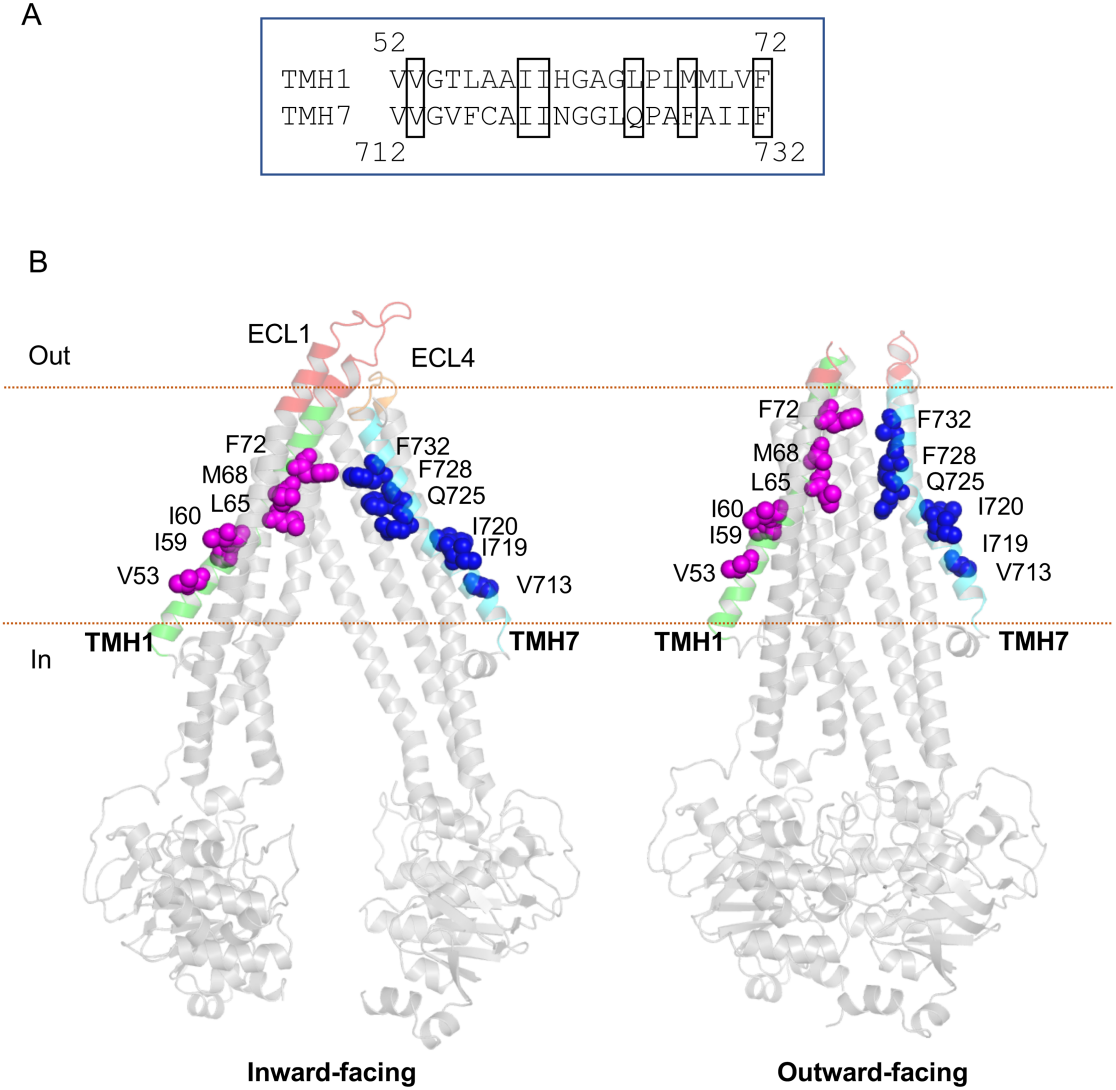
Location of TMH1 and TMH7 residues selected for mutagenesis. (A) Alignment of sequences of TMH1 and TMH7 of human P-gp with mutated residues shown in the box. (B) Location of TMH1 and TMH7 in a homology model of human P-gp derived from mouse P-gp (PDB.5KPI) in the inward-facing conformation (left) and the structure of the human P-gp E-Q mutant in the presence of ATP (PDB.6C0V) in the outward-facing conformation (right). TMH1 and TMH7 are colored green and cyan, respectively. The residues selected for mutation in TMH1,7 are shown as magenta (TMH1) and blue (TMH7) spheres. ECL1 and ECL4 are highlighted in red and orange, respectively. Cell membrane boundaries are marked. The figure was prepared in Pymol.

In the drug-binding pocket of P-gp, there are at least forty residues known to interact with substrates, based on earlier mutagenesis studies, modeling and co-crystallization data of mouse P-gp [16, 24–27, 32, 33, 45–49]. For our mutagenesis studies, we selected six residues each in TMH1 and TMH7 that are homologous to each other in sequence when aligned. These include V53, I59, I60, L65, M68, F72 in TMH1 and V713, I719, I720, Q725, F728, F732 in TMH7 (Fig 1A). The conservation of selected residues in human P-gp was assessed by using multiple sequence alignment of P-gp sequences from several mammalian species including those of the chimpanzee, monkey, dog, sheep, camel, cattle, pig, hamster, mouse and rat. The selected residues were found to be highly conserved among mammalian species (Table 1). When designing the mutations, we also considered other factors besides their conservation. We selected: (1) residues in TMH1 with homologous residues in TMH7; (2) residues that lined the drug-binding pocket; and (3) amino acids other than glycine or proline to avoid structural alterations in the helices caused by substitution of those residues.

**Table 1:** Conservation of residues selected for generation of TMH1,7 mutant P-gp across mammalian species.

Mammalian species	Residues selected for mutation in human ABCB1											
	VAL 53	ILE 59	ILE 60	LEU 65	MET 68	PHE 72	VAL 713	ILE 719	ILE 720	GLN 725	PHE 728	PHE 732
Chimpanzee ABCB1	●	●	●	●	●	●	●	●	●	●	●	●
Cynomolgus monkey ABCB1	●	●	●	●	●	●	●	●	●	●	●	●
Rhesus monkey ABCB1	●	●	●	●	●	●	●	●	●	●	●	●
Dog ABCB1	●	●	●	●	●	●	●	●	●	●	●	●
Camel ABCB1		●	●	●	●	●	●	●	●	●	●	●
*Pteropus alecto* (Bat) ABCB1		●	●	●	●	●	●		●	●	●	●
Pig ABCB1		●	●	●	●	●	●	●	●	●	●	●
*Myotis davidii* (Bat) ABCB1		●	●	●	●	●	●	●	●	●	●	●
Sheep ABCB1		●	●	●	●	●	●	●	●	●	●	●
Chinese hamster ABCB1a	●	●	●	●	●	●	●	●		●	●	●
Mouse ABCB1a	●	●	●	●	●	●	●	●	●	●	●	●
Rat ABCB1a		●	●	●		●	●		●	●	●	●
Rabbit ABCB1	●	●	●	●	●	●	●	●	●	●	●	●
Cattle ABCB1		●	●	●	●	●	●	●	●	●	●	●
Guinea Pig ABCB1		●	●	●		●	●	●	●	●	●	●
Chinese hamster ABCB1b				●		●	●		●	●	●	●
Rat ABCB1b		●	●	●		●	●		●	●	●	●
Mouse ABCB1b		●	●	●		●	●		●	●	●	●

The sequences of P-gp proteins from different species were retrieved from NCBI (https://www.ncbi.nlm.nih.gov/protein/).

Multiple sequence alignment was done by using ClustalOmega (https://www.ebi.ac.uk/Tools/msa/clustalo/).

The high-resolution structure of human P-gp in an inward-facing conformation is currently unavailable. Thus, a homology model for human P-gp was generated using the sequence of human P-gp and the mouse P-gp structure (PDB.5KPI, [21]) as a template. In the inward-facing conformation, TMH1 and TMH7 are aligned at a wider angle, with the cytosolic ends of the TMs at the periphery. In the outward-facing conformation (ATP-bound structure of E-Q mutant of human P-gp, PDB.6C0V, [22]), there seems to be a significant shift in the location of these residues. They are closer to the lumen of the cavity and form a narrower angle (Fig 1B). To understand the role of TMH1 and TMH7 in the P-gp transport cycle, we substituted selected residues with alanine and synthesized TMH1,7 mutant P-gp using GeneArt gene synthesis. For both WT and TMH1,7 mutant P-gp, we generated BacMam baculovirus for expression in HeLa and insect (High-Five) cells.

### TMH1,7 mutant P-gp with twelve substitutions is expressed to similar levels as WT P-gp in HeLa cells

**Fig 2:**
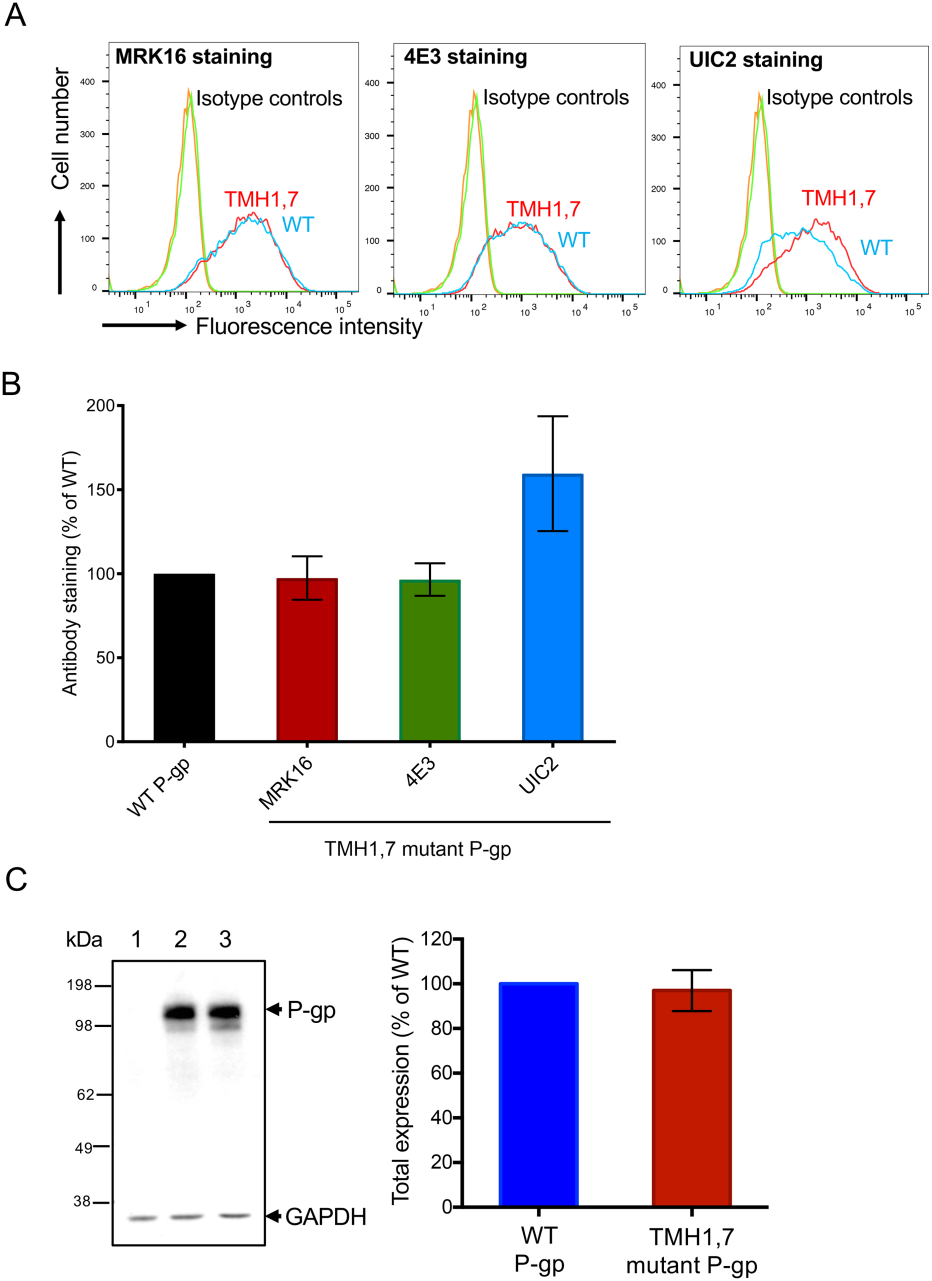
Total and cell surface expression of TMH1,7 mutant P-gp in HeLa cells. HeLa cell surface expression of TMH1,7 mutant P-gp was determined by staining with monoclonal antibodies against human P-gp: MRK16, 4E3 and UIC2. (A) In flow cytometry histograms, the curves show the expression of TMH1,7 (red) and WT P-gp (blue). IgG2aκ isotype controls are also shown. (B) Using surface expression data from flow-cytometric analysis in (A) and expression of WT P-gp taken as 100%, the relative expression of TMH1,7 mutant P-gp was calculated. Five to eight independent replicates were quantified, and error bars show SD. (C) Western blot of lysates of HeLa cells transduced with BacMam baculovirus showing the total expression of WT and TMH1,7 mutant P-gp using C219 antibody. The lysate of 60,000 untransduced cells (lane 1), cells expressing WT-P-gp (lane 2) or TMH1,7 mutant P-gp (lane 3) was loaded. The GAPDH expression was used as a loading control. The experiment was repeated three times with independent transductions and relative expression of TMH1,7 mutant P-gp was calculated using WT level as 100%, shown in bar graph (right). Error bar shows SD of three experiments.

P-gp is comprised of twelve TMHs joined by six ECLs. ECLs 1 and 4 contain the epitopes for the human P-gp-specific monoclonal antibodies MRK16, 4E3 and UIC2 that recognize specific conformations [50–53]. ECL1 is located between TMH1 and TMH2 and ECL4 is located between TMH7 and TMH8 (Fig 1B). To understand the possible effect of mutations in TMH1,7 on the conformation of ECLs1 and 4, WT and TMH1,7 mutant P-gp were expressed in HeLa cells. The cell surface expression of TMH1,7 mutant P-gp was compared with WT P-gp using all three antibodies by flow cytometry. As shown in Fig 2A and 2B, all three antibodies were able to bind to TMH1,7 mutant P-gp to the similar level to WT P-gp. However, UIC2 binding to the mutant protein was quite variable, ranging from 110–180% compared to WT protein. The total expression of WT and TMH1,7 mutant P-gp in HeLa cells was determined in cell lysates by Western blotting using the P-gp-specific antibody C219. The total expression of TMH1,7 mutant P-gp was also similar to that of WT protein in HeLa cells (Fig 2C). These results show that twelve mutations in TMH1 and TMH7 do not affect the overall expression or conformation of mutant P-gp in HeLa cells.

### TMH1,7 mutant P-gp cannot transport most of the substrates

We chose 25 fluorescent substrates that are structurally and functionally diverse amphipathic compounds ranging in size from 300–1500 Da to characterize the transport function of TMH1,7 mutant P-gp in HeLa cells. Most of the fluorescent compounds we tested were already established [54], while some had not previously been characterized as P-gp substrates. We used intrinsically fluorescent compounds such as daunorubicin, rhodamine 123, and DiOC_2_. Others were BODIPY-FL (BD) conjugates of compounds such as verapamil, vinblastine and prazosin. Ten substrates were derivatives of rhodamine (rhodamine 123, dihydrorhodamine 123, TMR-Cl, TMRE, rhodamine 6G, dihydrorhodamine 6G, Rhod-2-AM, X-Rhod-1-AM, rhodamine B hexyl ester and TMRM). Seven compounds were tested for the first time including azide-fluor 545, ER Tracker red, Mito-Tracker deep red FM, rhodamine B hexyl ester, Rhod-2-AM, X-Rhod-1-AM and TMRM. (A detailed characterization of the properties of these newly identified P-gp substrates will be discussed in a future study).

**Fig 3:**
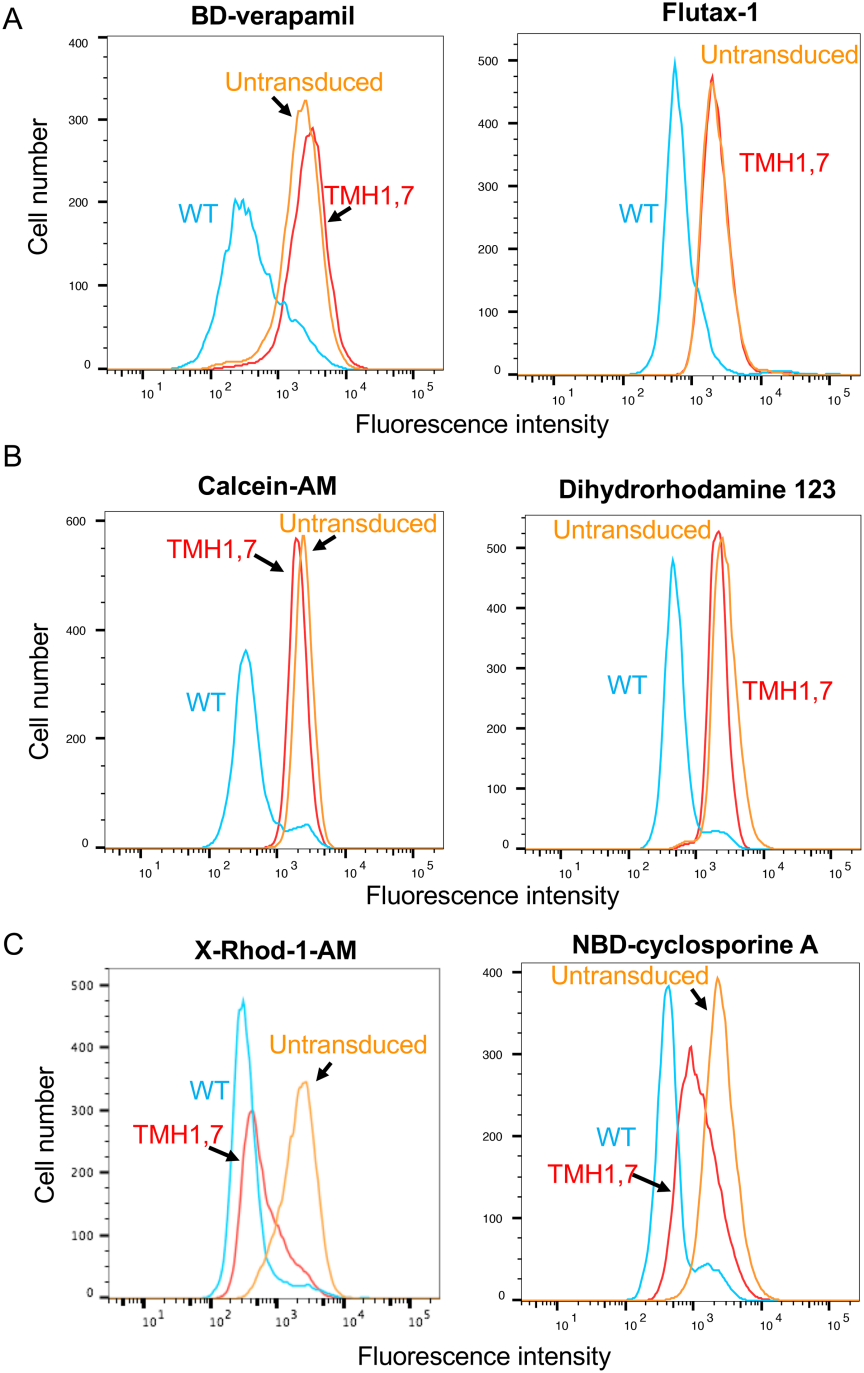
Characterization of the transport function of TMH1,7 mutant P-gp. WT and TMH1,7 mutant P-gp were expressed on HeLa cells by the BacMam baculovirus-based transduction system. The transport of fluorescent substrates was analyzed using flow cytometry. Histograms indicate the transport of representative substrates that are not transported by TMH1,7 mutant P-gp, (A) BODIPY-verapamil (0.5 μM) and flutax-1 (5 μM); substrates that are partially transported (10–30% compared to WT) by TMH1,7 mutant P-gp, (B) calcein-AM (0.5 μM) and dihydrorhodamine 123 (1.3 μM); and substrates that are efficiently transported by TMH1,7 mutant P-gp, (C) X-Rhod-1-AM (0.5 μM) and NBD-cyclosporine A (0.5 μM). Fluorescence intensity of WT P-gp is shown in blue, TMH1,7 mutant P-gp as red and untransduced cells are orange traces in all histograms.

**Fig 4:**
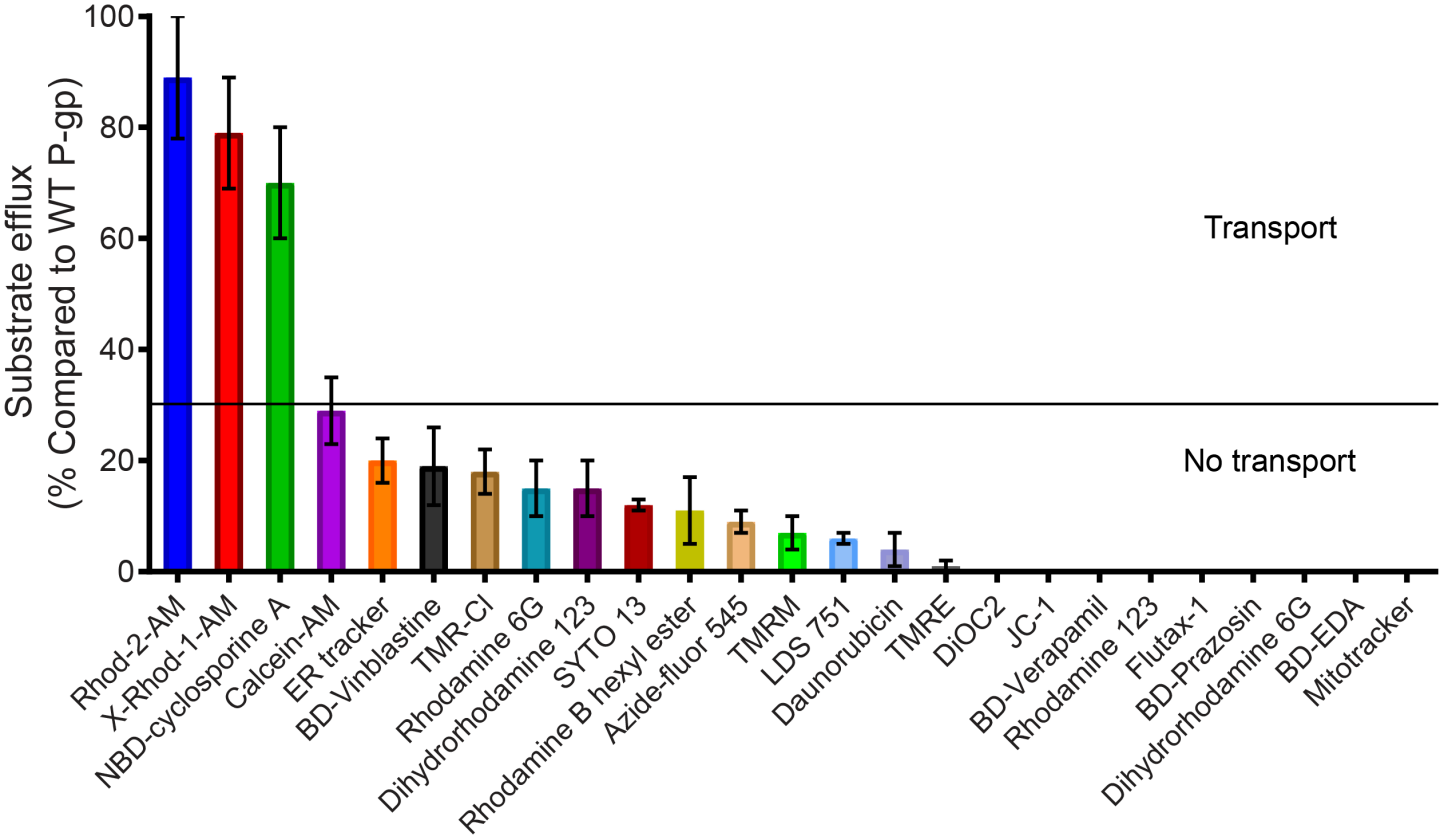
TMH1,7 mutant P-gp fails to transport most of the fluorescent substrates tested. WT and TMH1,7 mutant P-gp were expressed in HeLa cells by the BacMam baculovirus-based transduction system and 25 fluorescent substrates were used to determine their transport function. The efflux by WT P-gp (calculated as the value obtained after subtracting the mean fluorescence intensity of WT from untransduced cells) was taken as 100% and the efflux of indicated substrates by TMH1,7 mutant P-gp was calculated. The mean values from three to five independent experiments are shown and the error bars depict SD. The low level (<30%) was considered as no transport (solid line separating the two groups of substrates; see S1 Table for the level of transport of tested substrates by TMH1,7 mutant P-gp).

For the transport assays, untransduced HeLa cells not expressing detectable levels of P-gp were used as control (Fig 2C) and steady-state transport was measured. We found that TMH1,7 mutant P-gp was not able to efflux most of the substrates. The representative transport profiles of the substrates BD-verapamil and flutax-1, which are not transported by TMH1,7 mutant P-gp, are shown in Fig 3A. As shown in the histograms, the substrates were transported by WT (blue trace), while TMH1,7 mutant P-gp (red trace) failed to transport them (having similar fluorescence intensity as untransduced cells). The decreased fluorescence intensity of cells correlates with higher efflux and the presence of functional P-gp (WT, blue traces). The transport profiles of the substrates calcein-AM and dihydrorhodamine 123, which are partially transported (10–30% compared to WT P-gp), are shown in Fig 3B. For most of the substrates, the overall transport efficiency of TMH1,7 mutant P-gp was in the range of 0–30%, as compared to WT P-gp (taken as 100% efficiency) (Fig 4 and S1 Table).

### Three substrates are transported by TMH1,7 mutant P-gp

Among the 25 substrates tested, only three were efficiently transported by TMH1,7 mutant P-gp. Two of these were the rhodamine derivatives Rhod-2-AM (89% transport) and X-Rhod-1-AM (79% transport), and the third was NBD-cyclosporine A (70% transport) (their chemical structures are given in S1 Fig and results in Figs 3C and 4, and S1 Table). These results show that twelve mutations in P-gp did not lead to total loss of transport function, as it was still able to transport at least three substrates.

### TMH1,7 mutant P-gp exhibits lower basal ATPase activity

**Fig 5:**
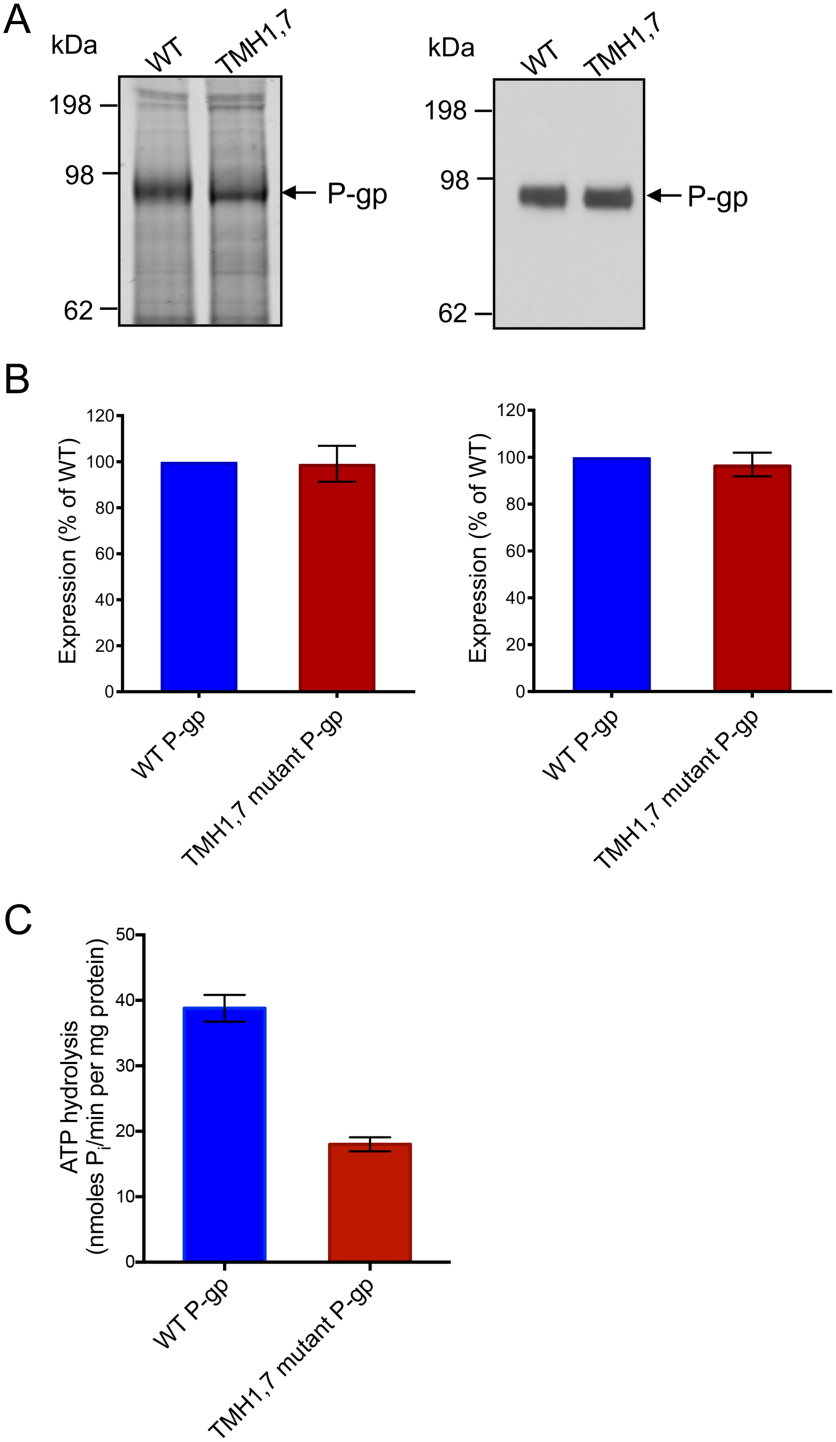
The basal ATPase activity of the TMH1,7 mutant is lower than WT P-gp despite similar expression in insect cell membranes. TMH1,7 mutant and WT P-gp membrane vesicles were prepared from High-Five insect cells by hypotonic lysis and differential centrifugation, as described in “Materials and Methods”. (A) 10 μg of total protein of membrane vesicles per lane was used for SDS-PAGE on 7% Tris-acetate gel (left) and 1 μg of total protein was used for Western blotting with C-219 antibody (right). (B) Bar graphs showing quantification of the SDS-PAGE gel using Licor Odyssey (left) and Western blot using Fiji-imageJ (right). The values represent the mean from three to five independent experiments and error bars show SD. (C) ATP hydrolysis was measured in the presence and absence of 0.3 mM sodium orthovanadate in membrane vesicles expressing WT or TMH1,7 mutant P-gp. Membranes (10 μg protein per 100 μL reaction volume) were incubated in ATPase assay buffer and vanadate-sensitive activity was measured as described previously [39]. The basal ATPase activity of TMH1,7 mutant P-gp was compared with WT P-gp. The histogram shows mean values from five independent assays in duplicate and error bars indicate SD.

Next, we studied the ATPase activity of WT and TMH1,7 mutant P-gp using membrane vesicles prepared from High Five insect cells. As for HeLa cells, the expression of TMH1,7 mutant P-gp in insect cells was comparable to that of WT protein, as confirmed by quantification of colloidal blue stained P-gp bands in 7% Tris-acetate gels and the chemiluminescence signal in immunoblots with C219 antibody (Fig 5A and 5B). ATPase assays were performed to determine the basal activity of WT and TMH1,7 mutant P-gp. The vanadate-sensitive basal ATPase activity of TMH1,7 mutant P-gp was found to be lower (18.0 ± 1.1 nmoles P_i_/min per mg protein) than that of WT protein (38.8 ± 2.0 nmoles P_i_/min per mg protein) (Fig 5C).

### P-gp substrates do not modulate the ATPase activity of the TMH1,7 mutant

**Fig 6:**
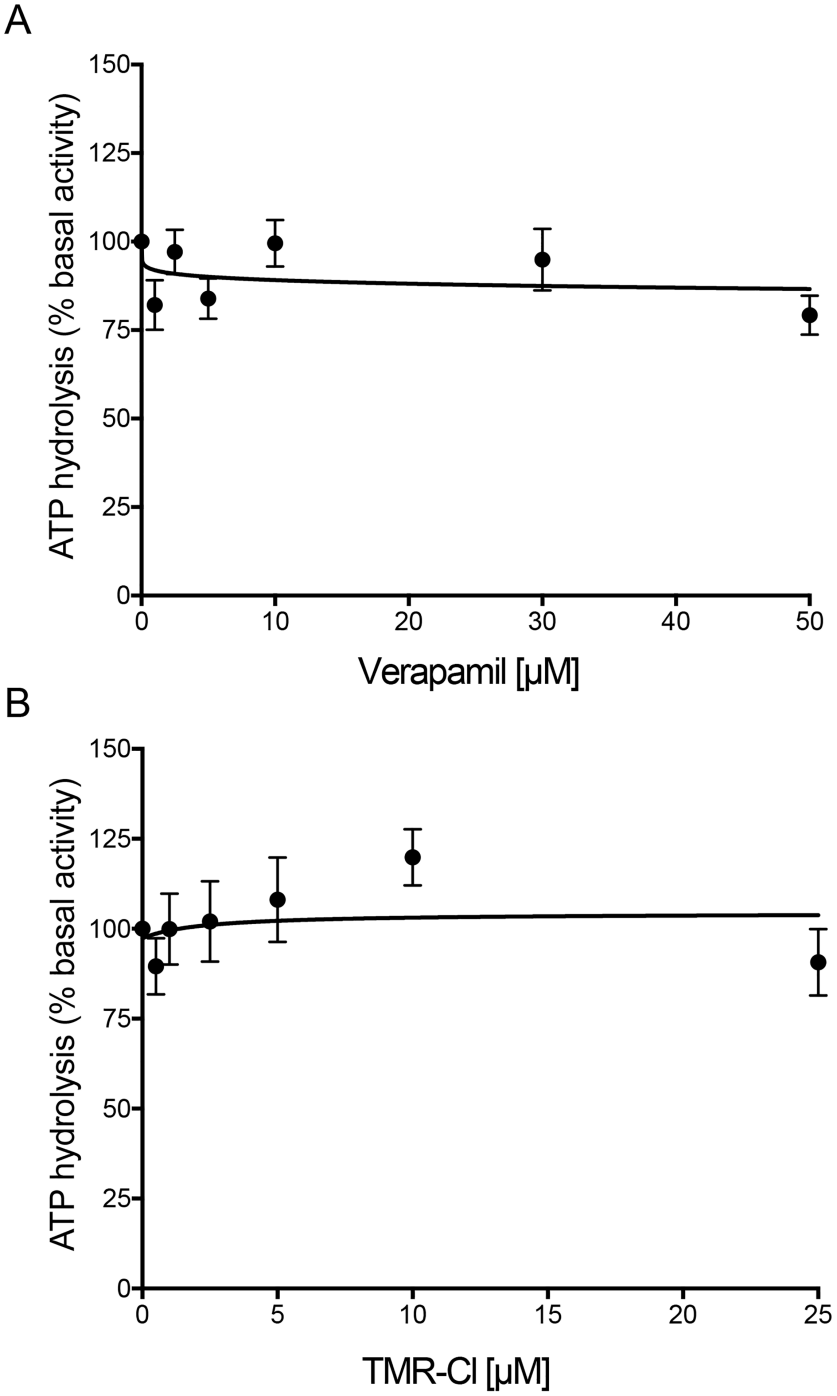
Verapamil and TMR-Cl do not stimulate the ATPase activity of TMH1,7 mutant P-gp. The effect of (A) verapamil and (B) TMR-Cl was tested on the ATPase activity of TMH1,7 and compared with WT P-gp [16]. The graphs show vanadate-sensitive ATPase activity of TMH1,7 in the absence of a substrate (basal activity) as 100% and relative activity calculated with increasing concentrations of verapamil (1–50 μM) and TMR-Cl (0.5–25 μM). The data points represent mean values from three independent experiments and error bars depict SD.

The ATPase activity of WT P-gp is stimulated by some of its transport substrates [15–17]. To test whether substrates stimulate the activity of TMH1,7 mutant P-gp, we performed ATPase assays in the presence of verapamil (1–50 μM) and TMR-Cl (0.5–25 μM), which are known to stimulate the activity of WT P-gp [16]. We did not observe any stimulation of TMH1,7 mutant P-gp ATPase activity in the presence of either verapamil or TMR-Cl (Fig 6).

**Fig 7:**
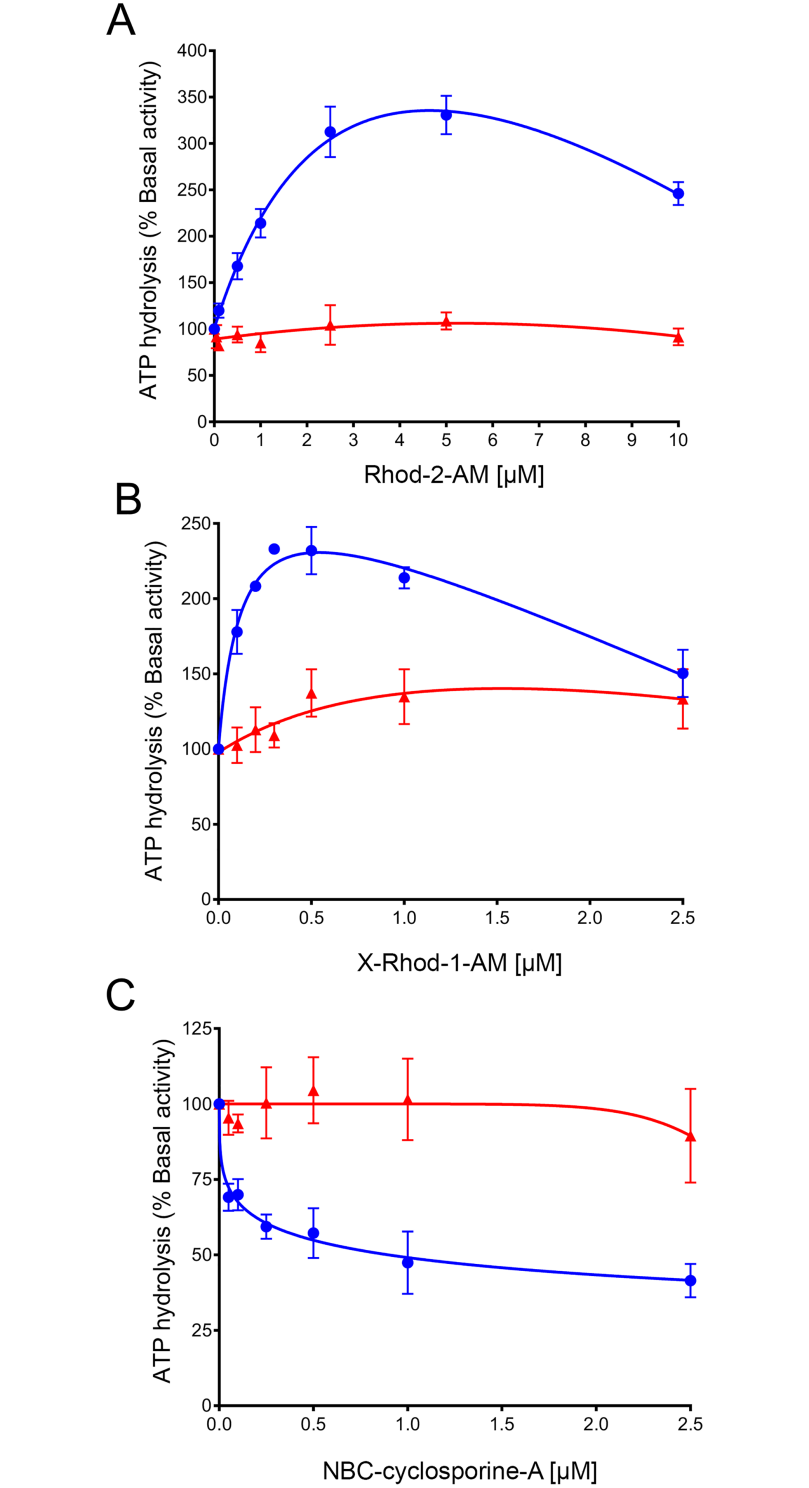
Effect of transported substrates on ATPase activity of TMH1,7 mutant P-gp. The vanadate-sensitive ATPase activity of TMH1,7 mutant P-gp was measured in the presence of three transported substrates. Graphs show the effect of (A) Rhod-2-AM (0.05–10 μM), (B) X-Rhod-1-AM (0.1–2.5 μM) and (C) NBD-cyclosporine A (0.05–2.5 μM), on the activity of WT (blue filled circles) and TMH1,7 mutant P-gp (red filled triangles). The mean values from three (A), seven (B) and six (C) independent experiments are given and error bars show SD.

Since both verapamil and TMR-Cl are not transported by TMH1,7 mutant P-gp, we investigated whether two of the substrates that are transported by the mutant (Rhod-2-AM and X-Rhod-1-AM) would stimulate its ATPase activity. We found that Rhod-2-AM stimulated WT ATPase by 3 to 4-fold at 5 μM (Fig 7A, blue filled circles). However, there was no effect on the activity of TMH1,7 mutant P-gp (Fig 7A, red filled triangles). X-Rhod-1-AM also stimulated WT P-gp ATPase activity by 2 to 3-fold (Fig 7B, blue filled circles), with maximum stimulation at 0.5 μM. At this concentration, there was stimulation ranging from 20 to 40% of TMH1,7 mutant P-gp ATPase activity (Fig 7B, red filled triangles). This result shows that the substrate-mediated stimulation of the ATPase activity of TMH1,7 mutant P-gp does not appear to be dependent on its ability to transport the given substrate. We also tested the effect of NBD-cyclosporine A on the ATPase of WT and TMH1,7 mutant P-gp. As shown in Fig 7C, NBD-cyclosporine A (0.05–2.5 μM) partially (40–50%) inhibited the activity of WT, while there was no significant effect on the ATPase activity of TMH1,7 mutant P-gp.

### *In silico* analysis of conformations of TMH1,7 mutant P-gp by homology modeling and docking of ligands

To gain insights into the loss of transport by TMH1,7 mutant P-gp, we performed *in silico* analysis using homology models of WT and TMH1,7 mutant P-gp generated by using the structure of mouse P-gp (PDB.5KPI) for the inward-facing conformation and the human P-gp E-Q mutant bound with ATP (PDB.6C0V) for the outward-facing conformation. While comparing both the conformations with WT P-gp, we observed that the amino acids in TMH1 and TMH7 have significant movement from one conformation to the other, with the region proximal to the cytosol having the maximum change. While measuring the distances between the corresponding pairs of amino acid residues in TMH1 and TMH7, we observed that the distance between residues (the distance calculated between α-C of each residue) F32 and F732 changes by approximately 1 Å, whereas between residues V53 and V713, it changes by 12 Å (S2 Fig). On aligning TMH1 and TMH7 in the inward-facing and outward-facing structures of the WT P-gp homology model, we observed a symmetrical bending of both the helices in the middle-axis that lead to decreased inter-helix distances (S3 Fig).

**Fig 8:**
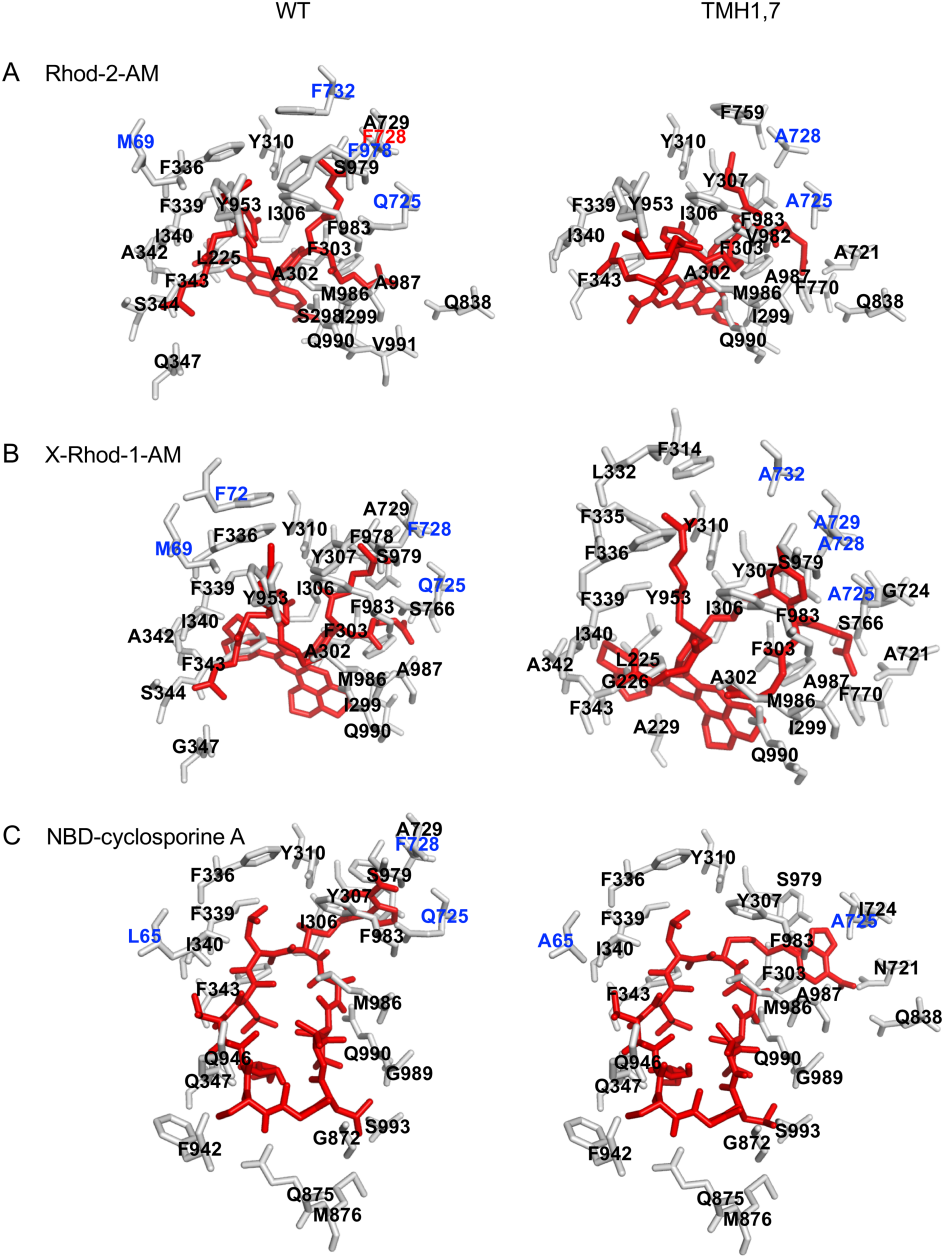
Docking of Rhod-2-AM, X-Rhod-1-AM and NBD-cyclosporine A in the drug-binding pocket of WT P-gp and TMH1,7. Homology models of human WT and TMH1,7 mutant P-gp (based on the PDB.5KPI mouse structure) were used for exhaustive ligand docking. The receptor grid was centered approximately at the position of the QZ59-RRR molecule (PDB.4M2S, [19]), and flexible side chains were defined based on proximity to this ligand. The amino acid residues mutated in TMH1,7 mutant P-gp were also set as flexible. The box size was defined as 70x40x40 Å. The lowest docking energy pose binding modes of Rhod-2-AM (A), X-Rhod-1-AM (B) and NBD-cyclosporine A (C) are presented. Rhod-2-AM, X-Rhod-1-AM and NBD-cyclosporine A are shown in red sticks. WT and TMH1,7 residues within 4Å distance of the ligand are presented in gray. The residues mutated in TMH1,7 mutant P-gp are highlighted in blue.

Subsequently, to address the loss of transport of many substrates by TMH1,7 mutant P-gp, we docked the three transported substrates in the homology models of both WT and TMH1,7 mutant P-gp in the inward-facing conformation. We used Autodock Vina, where the side chains of the mutated amino acids as well as the side chains of amino acids known to interact with cyclic peptide inhibitors QZ59-RRR and QZ59-SSS in the mouse P-gp structure [19] were set as flexible side-chains for docking. The docking of three substrates that are transported by the TMH1,7 mutant (S4 Fig) shows that these substrates bind in the drug-binding pocket and nine poses overlap with each other for a given ligand. The docking scores for nine poses of indicated ligands are presented in S2 Table. We observed that the docking scores of various substrates with TMH1,7 mutant P-gp were comparable to the WT protein, independently of whether they are transported or not by TMH1,7 mutant P-gp. However, analyses of the lowest energy docking poses of each substrate for TMH1,7 mutant and WT P-gp showed that even though in both cases the substrates bind in the drug-binding pocket in the transmembrane region, the interactions with specific residues were different for the WT and the mutant protein. This indicates that the ligand binding interactions were affected by the mutations. Fig 8 highlights the amino acid residues of both WT and TMH1,7 mutant P-gp that are within 4 Å distance in the lowest energy pose (pose 1 from S2 Table) for a given substrate for both.

## Discussion

Cancer cell lines become resistant to anti-cancer drugs by over-expressing P-gp. Due to its polyspecificity, P-gp can bind and transport a wide variety of cytotoxic agents out of the cell. In the past, we have generated P-gp mutants to understand the role of different sites within the drug-binding pocket [16, 17, 28]. In this study, we focused on a unique approach for generation of a P-gp mutant involving two homologous TM regions (TMH1 and TMH7) that exhibit topological symmetry in TMD1 and TMD2 (Fig 1). The expression profile of TMH1,7 mutant P-gp on the surface of HeLa cells was studied using monoclonal antibodies that recognize a specific conformation of the protein [50–53]. We observed that even after twelve mutations, the HeLa cell surface expression and localization was not altered. All three antibodies- MRK16, UIC2 and 4E3 recognized the TMH1,7 mutant, demonstrating that the overall conformation of the mutant was not altered to a detectable level due to mutations. Characterization of the transport function of TMH1,7 mutant P-gp revealed that it had lost the ability to transport 22 out of 25 substrates (Figs 3 and 4 and S1 Table). We became interested in understanding which of the steps during transport might be affected due to mutations. Molecular docking shows that the three substrates that were transported by the TMH1,7 mutant were able to bind in the drug-binding pocket with comparable docking scores to WT P-gp. Despite that, the direct interactions between substrates and the amino-acids in the drug-binding pocket were altered (Fig 8), suggesting that the substrate binding is affected by the mutations. This is consistent with the lack of significant effect of the transported substrates on the ATPase activity of TMH1,7 mutant (Fig 7). It is important to note that some of the residues located in the lower leaflet of the membrane might affect the communication between the substrate-binding pocket and the NBDs. It is possible that the twelve mutations could also interfere with the translocation step in the transport cycle, leading to the observed loss of transport phenotype. However, further studies will be required to verify this hypothesis.

To further understand why only three substrates were transported by the TMH1,7 mutant P-gp, we compared the physical, chemical and structural properties of the transported substrates with those of the non-transported substrates. However, we could not find any correlation between substrate size and its transport (although the molecular weight of transported substrates ranged between 1100 to 1350 Daltons, other substrates such as flutax-1, BD-vinblastine and calcein-AM, with similar size were not transported (S1 Table). There was no correlation between overall charge, polarity, number of hydrogen donor/acceptors or surface volume and the ability of TMH1,7 mutant P-gp to transport the substrates. It is important to note that the basal ATPase activity of the TMH1,7 mutant in membrane vesicles was lower than that of WT, but not completely lost (Fig 5). Furthermore, there was no significant effect on the vanadate-sensitive ATPase activity of the TMH1,7 mutant by substrates that can normally modulate the activity of WT P-gp. At present we can only speculate that mutations in TMH 1 and 7 affect the modulation of ATPase activity by altering the drug-binding and possibly the communication between substrate-binding sites and the NBDs.

Earlier studies using conformational dynamics and cross-linking of residues of P-gp in the inward-facing conformation revealed that the residues of TMH1 and TMH7 in the upper leaflet are more exposed to solvent than the rest of the transmembrane region and are involved in mediating conformational changes between the two states of P-gp [45, 55, 56]. In our study, the mutations in TMH1 and TMH7 occur throughout the transmembrane region (Fig 1). When the inward-facing and outward-facing conformations are compared, we observed that the greatest conformational change in these two helices occurs towards the cytosolic region, where the helices come closer together. In addition, there is a kink due to prolines 66 and 726 in TMH1 and TMH7, respectively, that becomes more pronounced while transitioning from the inward-facing to the outward-facing conformations, affecting the orientation of the amino acids in the central cavity of the binding-pocket. This indicates that residues in TMH1 and 7 selected for substitution modulate the drug-binding and transport.

In conclusion, we have characterized the role of TMH1 and TMH7 of human P-gp in the transport of several fluorescent substrates. This study highlights the extent of flexibility in the transmembrane domains, as twelve substitutions are well tolerated by P-gp, maintaining its conformation and level of expression similar to that of the WT at the cell surface. This is the first report to our knowledge demonstrating generation of a P-gp variant with a loss of broad substrate specificity. We observed loss of transport function for a majority (twenty-two out of twenty-five) of the tested substrates. Similar extensive alterations of other symmetrical paired helices such as TMH 2 and 8, TMH 5 and 11 or TMH 6 and 12 might provide useful information about the basis for the polyspecificity of the multidrug transporter.

## Supporting information

S1 Fig The chemical structures of transported substrates by TMH1,7 mutant P-gp. (PDF)

S2 Fig Distances between homologous residues mutated in TMHs 1 and 7. (PDF)

S3 Fig Topological arrangement of TMH1 and TMH7 in inward-facing and outward-facing conformations in WT P-gp. (PDF)

S4 Fig Docking of the transported substrates in the drug-binding pocket of the WT and TMH1,7 mutant P-gp in the inward-facing conformation. (PDF)

S1 Table Summary of transport of fluorescent substrates by TMH1,7 mutant P-gp. (DOCX)

S2 Table Docking scores of selected ligands in a homology model of WT and TMH1,7 mutant P-gp. (DOCX)
