# Supplementary material for: Evidence for the critical role of transmembrane helices 1 and 7 in substrate transport by human P-glycoprotein (ABCB1)
Source: PLoS One. 2018 Sep 28;13(9):e0204693. doi: 10.1371/journal.pone.0204693 (PMC6161881; doi:10.1371/journal.pone.0204693)
Supplement: S1 Fig — (PDF) [file pone.0204693.s001.pdf]

A

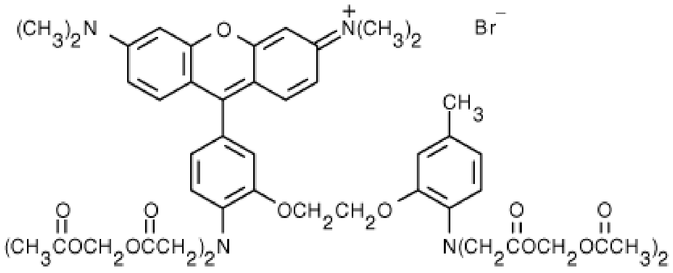

Rhod-2 AM Mol. Wt. 1124

B

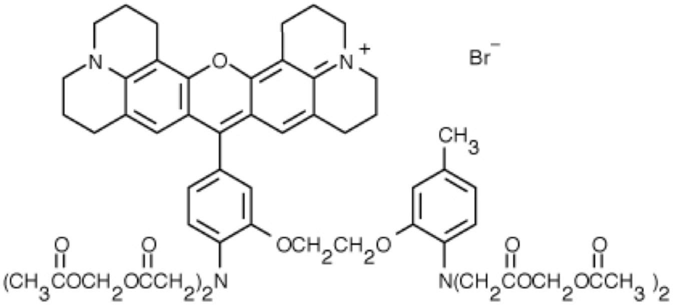

X-Rhod-1 AM Mol. Wt. 1228

C

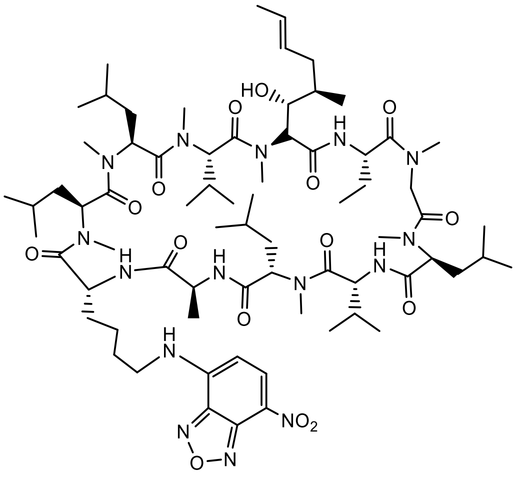

NBD-cyclosporine A Mol. Wt. 1422

S1 Figure. The chemical structures of transported substrates by TMH1,7 mutant P-gp. Chemical structures and molecular weights of the substrates that are transported by TMH1,7 mutant P-gp. (A) Rhod-2-AM, (B) X-Rhod-1-AM and (C) NBD-cyclosporine A.
