# Supplementary material for: Evidence for the critical role of transmembrane helices 1 and 7 in substrate transport by human P-glycoprotein (ABCB1)
Source: PLoS One. 2018 Sep 28;13(9):e0204693. doi: 10.1371/journal.pone.0204693 (PMC6161881; doi:10.1371/journal.pone.0204693)
Supplement: S2 Fig — (PDF) [file pone.0204693.s002.pdf]

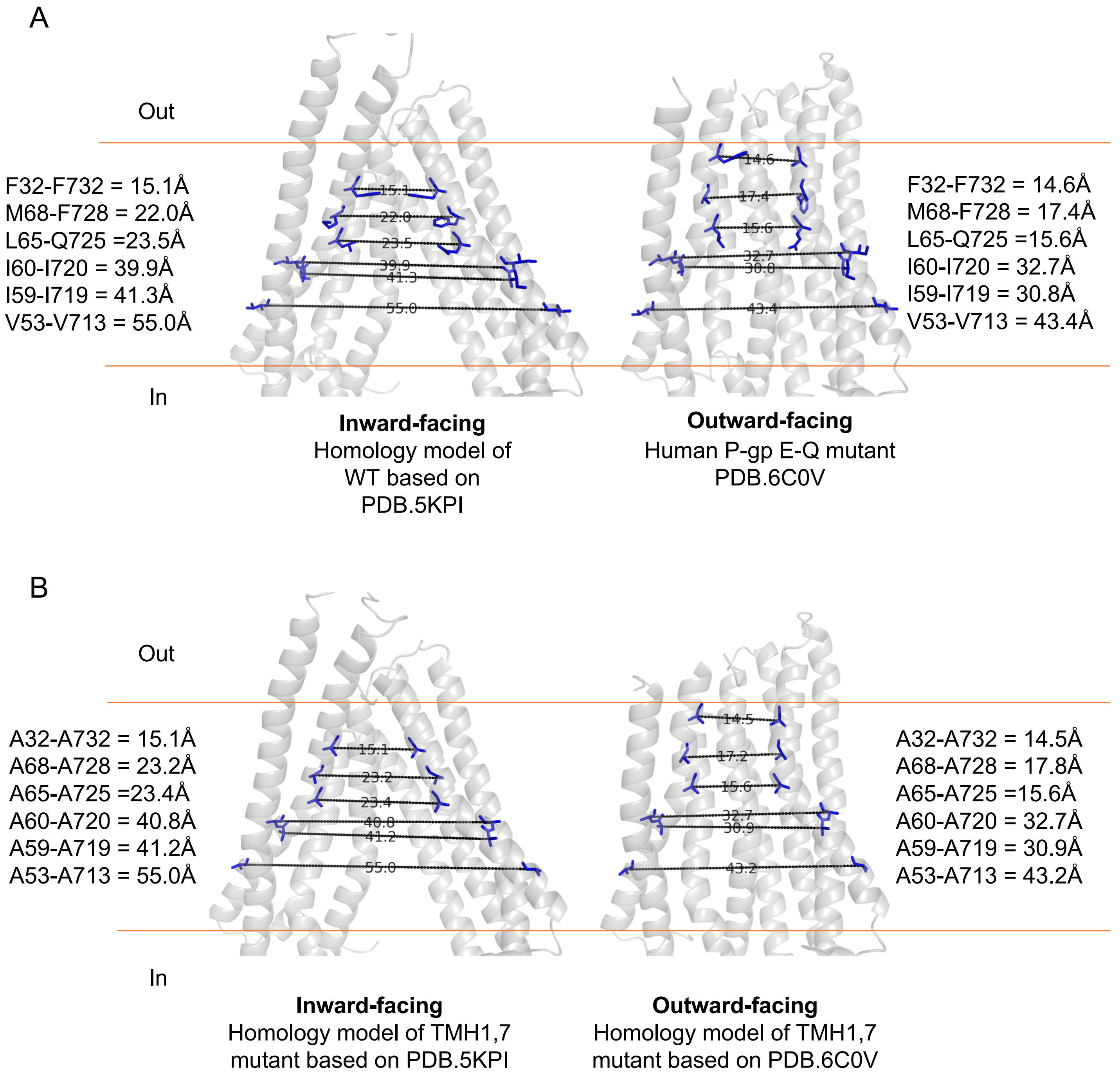

S2 Figure. Distances between homologous residues mutated in TMHs 1 and 7

Homology model of human (A) WT and (B) TMH1,7mutant P-gp in inward-facing (based on crystal structure of mouse P-gp; PDB.5KPI) and outward-facing conformations (cryo-EM structure of human P-gp E-Q mutant; PDB.6C0V). Amino acids mutated to alanine in the present study are highlighted by blue sticks. Residues 833-955 (which include TMH9-12 and ECL5 and 6) were removed for clarity. Distances between homologous amino acid pairs in WT (A) and mutated residues in TMH1,7mutant (B) are shown. Distances were measured between the α-carbons of the two amino acid residues. Residues closer to the inner leaflet show the greatest change in distance between two conformations. The figure was prepared in Pymol (version 7).
