# Supplementary material for: Evidence for the critical role of transmembrane helices 1 and 7 in substrate transport by human P-glycoprotein (ABCB1)
Source: PLoS One. 2018 Sep 28;13(9):e0204693. doi: 10.1371/journal.pone.0204693 (PMC6161881; doi:10.1371/journal.pone.0204693)
Supplement: S3 Fig — (PDF) [file pone.0204693.s003.pdf]

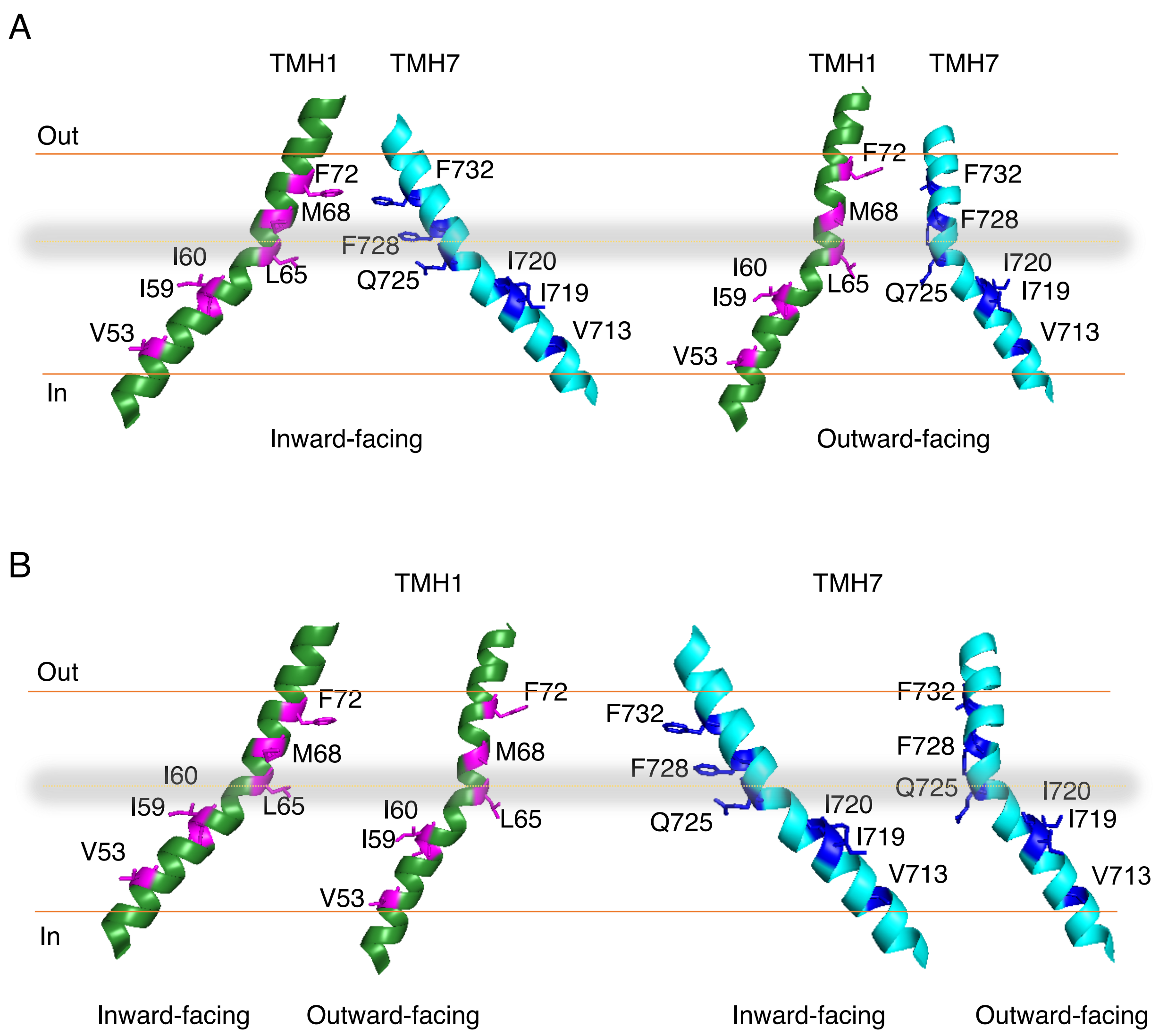

S3 Figure. Topological arrangement of TMH1 and TMH7 in inward-facing and outward-facing conformations in WT P-gp

A homology model of human P-gp was generated using the mouse P-gp structure (PDB.5KPI) for inward-facing conformation and the human E-Q mutant P-gp structure (PDB.6C0V) was used for the outward-facing conformation. TMH1 and TMH7 are shown with mutated residues highlighted in magenta for TMH1 and dark blue for TMH7 as sticks. (A) Comparison of TMH1 and TMH7 in inward-facing (left) and outward-facing (right) conformations. (B) Comparison of TMH1 (left) and TMH7 (right) in inward- and outward-facing conformations. The membrane boundaries are shown by orange lines and the central axis where the helices are bending after conformational change is highlighted as a grey region. The figures were prepared in Pymol (version 7).
