# Supplementary material for: Evidence for the critical role of transmembrane helices 1 and 7 in substrate transport by human P-glycoprotein (ABCB1)
Source: PLoS One. 2018 Sep 28;13(9):e0204693. doi: 10.1371/journal.pone.0204693 (PMC6161881; doi:10.1371/journal.pone.0204693)
Supplement: S4 Fig — (PDF) [file pone.0204693.s004.pdf]

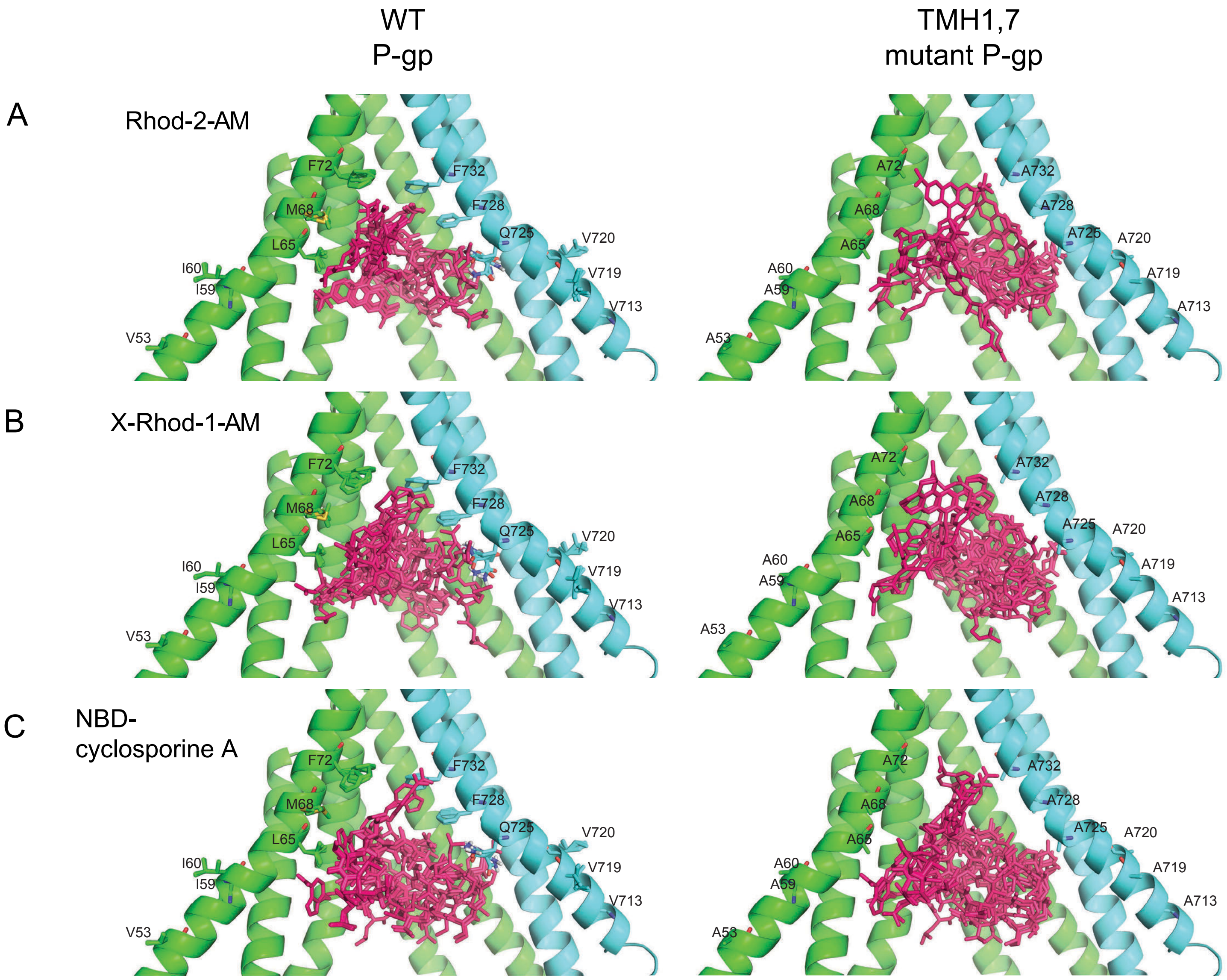

S4 Figure. Docking of the transported substrates in the drug-binding pocket of the WT and TMH1,7 mutant P-gp in the inward-facing conformation. Homology models of human WT and TMH1,7 mutant P-gp (based on the PDB.5KPI mouse structure) were used for exhaustive ligand docking. The receptor grid was centered approximately at the position of the QZ59-RRR molecule (PDB.4M2S, [19]), and side chains were defined based on proximity to this ligand. The residues mutated in TMH1,7 mutant P-gp were set as flexible. The box size was defined as 70x40x40 Å. The first nine poses with the highest docking scores were clustered and shown as magenta sticks for WT P-gp (left) and TMH1,7 mutant P-gp (right) with (A) Rhod-2-AM, (B) X-Rhod-1-AM, and (C) NBD-cyclosporine A. For clarity, TMHs 2, and 8-12 are not shown; the rest of the helices of TMD1 (green) and TMD2 (cyan) are presented as a cartoon. The figure was prepared in Pymol.
