# Supplementary material for: Evidence for the critical role of transmembrane helices 1 and 7 in substrate transport by human P-glycoprotein (ABCB1)
Source: PLoS One. 2018 Sep 28;13(9):e0204693. doi: 10.1371/journal.pone.0204693 (PMC6161881; doi:10.1371/journal.pone.0204693)
Supplement: S1 Table — (DOCX) [file pone.0204693.s005.docx]

**S1 Table. Summary of transport of fluorescent substrates by TMH1,7 mutant P-gp**

| **Substrate** | **Transport**  **(% of WT P-gp)*** |
| --- | --- |
| Rhod-2-AM | 89±11 |
| X-Rhod-1-AM | 79±10 |
| NBD-cyclosporine A | 70±10 |
| Calcein-AM | 29±6 |
| ER Tracker | 20±4 |
| BD-Vinblastine | 19±7 |
| TMR-Cl | 18±4 |
| Rhodamine 6G | 15±5 |
| Dihydrorhodamine 123 | 15±5 |
| SYTO-13 | 12±1 |
| Rhodamine B, hexyl ester | 11±6 |
| Azide-fluor 545 | 9±2 |
| TMRM | 7±3 |
| LDS-751 | 6±1 |
| Daunorubicin | 4±3 |
| TMRE | 1±1 |
| DiOC2 | Not detectable |
| JC-1 | Not detectable |
| BD-Verapamil | Not detectable |
| Rhodamine 123 | Not detectable |
| Flutax-1 | Not detectable |
| BD-Prazosin | Not detectable |
| Dihydrorhodamine 6G | Not detectable |
| BD-EDA | Not detectable |
| MitoTracker deep red FM | Not detectable |

*The transport assay was performed at least three times and values represent mean ± SD

Abbreviations: BODIPY (BD)-verapamil; BD-3-Propionyl ethylenediamine hydrochloride (BD-EDA); 3,3'-Diethyloxacarbocyanine iodide (DiOC_2_); Quinolinium, 6-(dimethylamino)-2-[4-[4-(dimethylamino)phenyl]-1,3-butadienyl]-1-ethyl, perchlorate (LDS-751); Tetramethylrosamine chloride (TMR-Cl); tetramethylrhodamine ethyl ester perchlorate (TMRE); Tetramethylrhodamine methyl ester perchlorate (TMRM).
