# Supplementary material for: Evidence for the critical role of transmembrane helices 1 and 7 in substrate transport by human P-glycoprotein (ABCB1)
Source: PLoS One. 2018 Sep 28;13(9):e0204693. doi: 10.1371/journal.pone.0204693 (PMC6161881; doi:10.1371/journal.pone.0204693)
Supplement: S2 Table — (DOCX) [file pone.0204693.s006.docx]

**S2 Table. Docking scores of selected ligands in a homology model of WT and TMH1,7 mutant P-gp.**

| Pose # | Rhod-2-AM | | X-Rhod-1-AM | | NBD-cyclosporine A | | Rhodamine 123 | | Flutax-1 | | BD-Verapamil | |
| --- | --- | --- | --- | --- | --- | --- | --- | --- | --- | --- | --- | --- |
|  | WT | TMH1,7 | WT | TMH1,7 | WT | TMH1,7 | WT | TMH1,7 | WT | TMH1,7 | WT | TMH1,7 |
| 1 | -10.1 | -9.1 | -11.8 | -11.1 | -11.8 | -11.0 | -8.9 | -9.7 | -13.0 | -14.2 | -11.5 | -11.4 |
| 2 | -10.1 | -8.9 | -11.4 | -11.0 | -11.6 | -11.0 | -8.7 | -9.7 | -12.9 | -14.1 | -11.3 | -10.9 |
| 3 | -10.1 | -8.9 | -11.2 | -10.9 | -11.5 | -11.0 | -8.7 | -9.6 | -12.9 | -13.8 | -11.3 | -10.8 |
| 4 | -10.0 | -8.9 | -11.1 | -10.9 | -11.4 | -10.9 | -8.7 | -9.6 | -12.8 | -13.7 | -11.1 | -10.7 |
| 5 | -10.0 | -8.8 | -11.1 | -10.8 | -11.2 | -10.9 | -8.6 | -9.6 | -12.7 | -13.6 | -11.1 | -10.7 |
| 6 | -9.9 | -8.8 | -11.1 | -10.8 | -11.1 | -10.7 | -8.6 | -9.2 | -12.7 | -13.5 | -11.0 | -10.6 |
| 7 | -9.9 | -8.8 | -11.1 | -10.8 | -11.1 | -10.6 | -8.4 | -9.0 | -12.6 | -13.5 | -11.0 | -10.6 |
| 8 | -9.8 | -8.8 | -11.1 | -10.8 | -11.0 | -10.6 | -8.4 | -8.9 | -12.6 | -13.5 | -10.9 | -10.6 |
| 9 | -9.8 | -8.8 | -11.0 | -10.8 | -11.0 | -10.6 | -8.4 | -8.9 | -12.6 | -13.5 | -10.9 | -10.4 |

Homology models of inward-open conformation of P-gp (WT and TMH1,7) were used for docking of selected substrates. Docking scores are presented in kcal/mol. For docking, the center of the receptor grid was set at x=20, y=55 and z=5. The inner box dimensions were 70x40x40 Å and the exhaustiveness level was set at 100. A total of 32 residues were set as flexible. Other details are given in the “Materials and Methods” section.
